# Characteristics of resting-state functional connectivity in older adults after the PICMOR intervention program: a preliminary report

**DOI:** 10.1186/s12877-020-01892-2

**Published:** 2020-11-20

**Authors:** Hikaru Sugimoto, Toshikazu Kawagoe, Mihoko Otake-Matsuura

**Affiliations:** 1 RIKEN Center for Advanced Intelligence Project, Nihonbashi 1-chome Mitsui Building, 15th floor, 1-4-1 Nihonbashi, Chuo-ku, Tokyo, 103-0027 Japan; 2 Department of Psychology, College of Contemporary Psychology, Rikkyo University, 1-2-26, Kitano, Niiza City, Saitama 352-8558 Japan

**Keywords:** Intervention, Left inferior frontal gyrus, PICMOR, Resting-state functional MRI, Temporal pole, Verbal fluency

## Abstract

**Background:**

The present study aimed to provide a basis for future research examining the neural mechanisms that underlie the beneficial effect of an intervention program, Photo-Integrated Conversation Moderated by Robots (PICMOR), on verbal fluency in older adults as identified in our previous randomized controlled trial. In this preliminary report, we conducted an additional experiment using resting-state functional magnetic resonance imaging (rsfMRI) after the intervention period. Specifically, we investigated the resting-state functional connectivity (rsFC) characteristics of the intervention group (INT) compared to the control group (CONT).

**Methods:**

rsfMRI data were acquired from 31 and 30 participants in INT and CONT, respectively, after the intervention. In the analyses, two of the most important regions in verbal fluency, the left inferior and middle frontal gyri, were selected as seed regions, and the rsFCs were compared between groups. We also conducted regression analyses for rsFCs using the difference in individual phonemic verbal fluency task (PVFT) scores between the pre- and post-intervention periods (i.e., post- minus pre-intervention) as an independent variable.

**Results:**

We found higher rsFC in INT than in CONT between the left inferior frontal gyrus as a seed region and the temporal pole and middle frontal gyrus. The rsFC strength between the left inferior frontal gyrus and temporal pole positively correlated with an increased PVFT score between the pre- and post-intervention periods. In contrast, we found lower rsFC in INT than in CONT between the left middle frontal gyrus as a seed region and the posterior cingulate cortex, precuneus, and postcentral gyrus.

**Conclusions:**

Our findings suggest that the beneficial intervention effect of PICMOR on verbal fluency is characterized by enhanced rsFC of the left inferior frontal gyrus with semantic and executive control-related regions and suppressed rsFC between the left middle frontal gyrus and posterior cortical midline structures. No definitive conclusions can be made because of a lack of rsfMRI data before the intervention. However, this pilot study provides the candidates for rsFCs, reflecting the beneficial effects of PICMOR on the brain network involved in verbal fluency.

**Trial registration:**

The trial was retrospectively registered at the UMIN Clinical Trials Registry (UMIN000036667) (May 7th, 2019).

**Supplementary Information:**

**Supplementary information** accompanies this paper at 10.1186/s12877-020-01892-2.

## Background

Interest in the development of methods to prevent or delay dementia is growing rapidly [1]. Given the evidence of a relationship between cognitive function and social interaction [2], interventions that include social activities could be candidates for such methods. However, there is still little evidence regarding the effects of social activity-based interventions on cognitive function [3]. Toward addressing this gap, we have developed an intervention program, Photo-Integrated Conversation Moderated by Robots (PICMOR) [4]. The PICMOR offers a moderated group conversation context with robot-based facilitation. Specifically, a robot encourages a participant to talk about their daily life, using photos they have prepared beforehand, and to answer questions from the other human participants. The other participants are required to listen carefully and ask the speaker questions. This program is partly characterized by enforced output, where the system and robot have been designed to directly and strongly encourage participants to talk about various topics for a certain length of time. To examine the effect of PICMOR on cognitive function in older adults, we conducted a randomized controlled trial (RCT) [4]. The main finding of our RCT was that there was a significantly larger increase in the phonemic verbal fluency task (PVFT) score, which is included in the Japanese version of the Montreal Cognitive Assessment [5], in the intervention group (INT) than in the control group (CONT) between the pre- and post-intervention periods. Therefore, it is reasonable to presume that this behavioral effect could be reflected in differences between the two groups in the brain network responsible for verbal fluency. To examine the differences between INT and CONT in the functional network of the brain regions involved in verbal fluency, we conducted an additional experiment after the intervention period using resting-state functional magnetic resonance imaging (rsfMRI), which has been associated with an individual’s ability to perform cognitive tasks [6], including phonemic fluency ability [7].

In this preliminary report, we performed a seed-based functional connectivity analysis on the rsfMRI data [8], which were obtained after the intervention period from participants in our previous RCT study. In the analyses, we employed the left inferior and middle frontal gyri as seed regions, including a classical language-related region, called Broca’s area [9–11]. These regions were selected because it has been suggested that they are the most important for verbal fluency. Indeed, previous neuropsychological studies have consistently shown that patients with lesions of the left frontal lobe have impaired phonemic verbal fluency [12–15]. In addition, previous neuroimaging meta-analyses have reported that the left inferior frontal gyrus and/or middle frontal gyrus consistently show significant activation during PVFTs [10, 11]. These findings suggest that the left inferior and middle frontal gyri play pivotal roles in phonemic verbal fluency. Thus, we examined how the resting-state functional connectivity (rsFC) with these seed regions differed between INT and CONT, assuming that the differences between the groups were associated with the relatively large enhancement of the PVFT score in INT compared to CONT [4]. The differences between INT and CONT cannot be entirely attributed to the effect of PICMOR because we lack comparable rsfMRI data from the pre-intervention period. Therefore, no definitive conclusions can be made from this preliminary report. However, this study provides the candidates for rsFCs, reflecting the beneficial intervention effects of PICMOR on the brain network involved in verbal fluency.

## Methods

### Participants

Sixty-five healthy community-dwelling older adults, who participated in our previous RCT study (32 and 33 participants in INT and CONT, respectively) [4], were recruited in the present study. The psycho-demographic data of these participants are detailed in our previous report [4]. A total of sixty-one participants, including 31 and 30 participants in INT and CONT, respectively, took part in MRI scanning. MRI data from four RCT participants were not collected because one and one participant in INT and CONT, respectively, had claustrophobia, one participant in CONT was equipped with a heart pacemaker, and one participant in CONT declined to undergo the MRI scanning. All participants were right-handed and native Japanese-speaking individuals. We confirmed that there were no significant differences in age (*t* [*df* = 59] = 1.01, *p* = 0.32), sex (*χ*^*2*^ [*df* = 1, *n* = 61] = 0.15, *p* = 0.70), and educational level (*χ*^*2*^ [*df* = 1, *n* = 61] = 0.13, *p* = 0.71) between the two groups (educational level was binarized with a border of 13 years to categorize participants depending on whether they went to university or college; see Table 1).

**Table 1:** Participants’ characteristics

	INT (***N*** = 31)	CONT (***N*** = 30)
Age (mean ± SD)	72.84 ± 3.45	72.03 ± 2.72
Sex (males: females)	16: 15	13: 17
Educational level (≥ 13 years: < 13 years)	20: 11	17: 13
Amount of talking time in group conversations during the intervention period** (sec) (mean ± SD)	5000.52 ± 800.63	7627.19 ± 3377.06
PVFT score at the pre-intervention (mean ± SD)	12.03 ± 3.61	11.13 ± 4.07
PVFT score at the post-intervention** (mean ± SD)	13.71 ± 3.55	11.07 ± 2.95

We lack conversational data of one out of 12 group conversations from two CONT groups due to technical errors. Asterisks represent the variables showing significant difference between INT and CONT (*p* < 0.01)

*Abbreviations*: *INT* the intervention group, *CONT* the control group, *PVFT* phonemic verbal fluency task, *SD* standard deviation

### Intervention program

The procedures used in the PICMOR intervention and control program are described in detail below. Both the intervention and control programs were based on a group conversation. The 65 people recruited from the Silver Human Resources Center were divided into 16 groups (eight and eight in INT and CONT, respectively), each with four members, except for one CONT, which had five members. The participants were required to participate in the group conversations once a week for 12 weeks. One of the major differences between the two programs was whether the programs were designed to train executive functions.

In the group conversation offered by PICMOR, a robot acted as the chair to lead the conversations and prompt one of the four members to speak about an event they had experienced in their daily life for 1 min. The topic was a predetermined subject that changed every week. During this period, the other three members of the group had to listen attentively so that they could ask questions during the discussion period. The 1 min talking period was repeated without a break, in which they talked about another event related to the topic (i.e., each participant was assigned a total of 2 min to talk). Following this, there was a 2 min discussion period for each event during which the speaker was required to answer questions raised by the other three members of the group. During the discussion periods, the robot automatically encouraged and stopped the participants’ utterances to balance the amount of talking time allocated to each participant. For example, when the robot detected that one participant had spent less time talking than others, it directly prompted the participant to comment or ask a question. After the 2 min discussion periods, another member was assigned as the speaker. This procedure (i.e., the 1 min talking periods, followed by the 2 min discussion periods) was repeated for all members. There were two major reasons for using a robot and not a human as the chair. First, we could force the participants to make a speech during the predetermined time and finish their talk when their allocated time was over, and ensure that uncontrollable personal factors that can arise from a human chair, such as hesitation, were excluded. Time management by the robot made it possible to give each member an equally predetermined time (i.e., 1 min) to talk about an event. Second, it would be challenging for a human chairperson to prompt and stop the conversations in real-time based on the talking time of each person.

In contrast, in the group conversations offered by the control program, four members were required to talk freely without any robotic facilitation or predetermined theme, similar to how they would converse in their daily life. As shown in Table 1, the variance of the amount of time spent talking in group conversations during the intervention period was smaller in INT than in CONT. In an *F*-test to compare the variances between the two groups, the null hypothesis (i.e., the true ratio of variance is 1) was rejected (ratio of variances = 17.79, *p* < 0.01). This suggests that our experimental manipulation by robotic moderation to balance the amount of talking time for each participant in INT was successful. We hypothesized that repeated training in group conversations in PICMOR compared to the control program would exercise executive functions, such as flexibility, planning, working memory, and response inhibition, given that the participants have to make a speech within a limited time (i.e., 1 min), flexibly ask and answer questions, intentionally store and manipulate information to ask questions, and suppress the interruption of other members in a group conversation. Executive control and verbal abilities can be measured using the verbal fluency task [16], in which participants are required to produce as many words as possible beginning with a specific letter (e.g., /ka/ in Japanese) [5]. The number of correct unique words generated in 1 min is often used as a measure of performance. To successfully perform this task, participants must flexibly retrieve appropriate words along with the task rules, from their long-term memory (semantic memory), which most likely involves accessing their mental lexicon [16–18]. Successful task performance also requires them to keep previously produced words in their working memory so as to avoid repetition and suppress inappropriate words or task-irrelevant thoughts, which may involve executive control processes [16–18]. Given that the characteristics of the training demands in PICMOR involve exercising executive functions, it is reasonable to predict that the ability to produce words within a limited time could be enhanced in INT compared with CONT between the pre- and post-intervention periods. Consistent with this idea, a significantly larger improvement in the PVFT score was observed in INT than in CONT in our previous RCT study [4]. The beneficial intervention effect on verbal fluency could not be explained by the amount of talking time in group conversations during the intervention period, given that the talking time was significantly shorter in INT than in CONT (*t* [*df* = 59] = 4.21, *p* < 0.01, *Cohen’s d* = 1.08; see Table 1).

### Data acquisition

All MRI data were acquired with a Philips Achieva 3.0 MRI scanner, located in the Advanced Imaging Center Yaesu Clinic, Tokyo. Data were collected only after the intervention. During the MRI scanning, participants were equipped with a set of earplugs and headphones to reduce the effects of scanner noise and a belt with foam pads around their head to minimize head motion. There was no significant difference in the time period from the last day of the intervention period to the day of MRI data acquisition between INT (mean ± SD = 9.67 ± 0.76 weeks) and CONT (mean ± SD = 9.68 ± 0.56 weeks) (*t* [*df* = 59] = 0.05, *p* = 0.96).

First, three directional T1-weighted anatomical planes were scanned to localize the subsequent anatomical and functional images. Subsequently, anatomical structures were scanned using a high-resolution T1-weighted image (repetition time [TR] = 6.41 ms, echo time [TE] = 3.00 ms, field of view [FOV] = 24.0 cm × 24.0 cm, matrix size = 256 × 256, slice thickness/gap = 1.2/0 mm, 170 sagittal slices). Finally, resting-state functional images were scanned using a pulse sequence of gradient-echo echo-planar imaging, which is sensitive to blood oxygenation level-dependent (BOLD) contrasts (TR = 3000 ms, TE = 30 ms, flip angle = 80 degrees, FOV = 24.0 cm × 24.0 cm, matrix size = 80 × 80, slice thickness/gap = 4.0/0 mm, 35 horizontal slices). All participants were instructed to remain awake with their eyes open and think of nothing during the entire rsfMRI scan (10 min). The rsfMRI run began with dummy scans that were discarded from further analyses.

### Data analysis

All MRI data were analyzed using the CONN functional connectivity toolbox v.17.f (www.nitrc.org/projects/conn) [19] for Statistical Parametric Mapping 12 (SPM 12) (www.fil.ion.ucl.ac.uk/spm/), implemented in MATLAB. Resting-state functional images were preprocessed along the default pipeline in CONN. The images were realigned and corrected for slice timing. After the outlier detection, the functional and structural images were segmented and normalized to the Montreal Neurological Institute (MNI) space with a resolution of 2 × 2 × 2 mm^3^ voxels. These normalized functional images were spatially smoothed by a Gaussian kernel of 8 mm full-width at half-maximum. Subsequently, temporal correction was performed using the component-based noise correction method [20]. In this step, five principal components were extracted from the white matter and cerebrospinal fluid regions. Along with six bulk motion parameters, the first-order derivative of each motion parameter, and the scrubbing parameter, the five principal components were regressed out from the signal of interest. The scrubbing parameter was provided using the Artifact Detection Tools in CONN that can detect outliers based on the variance of whole movements. A band-pass filter with a frequency window of 0.008–0.09 Hz and detrending were then applied to the data.

In the present study, we used seed-to-voxel analyses of the rsfMRI data. Seed regions were defined as spheres with a 5 mm radius, around (− 50, 12, 24), (− 48, 28, 14), (− 52, 12, 0), (− 42, 8, 36), (− 54, 2, 46), and (− 44, 18, 6) in the MNI coordinates, located in the left inferior and middle frontal gyri, based on a previous neuroimaging meta-analysis demonstrating that these regions consistently show significant activation during PVFTs [11]. The anatomical mask of these seed regions was created using MarsBaR (www.marsbar.sourceforge.net). In the individual-level analysis, the mean BOLD time course was extracted from each seed region, and correlation coefficients were calculated with the BOLD time course of each voxel, throughout the whole-brain. The coefficients were converted to normally distributed scores using Fisher’s transformation. This procedure yielded individual rsFC maps for each seed region. In the group-level analysis, the rsFC maps identified in the first level analysis of INT and CONT were compared using two-sample *t*-tests. The models included age, sex, and educational level as covariates. In this second-level analysis, the threshold at the cluster level was corrected for whole-brain multiple comparisons (false discovery rate [FDR]; *p* < 0.05). Given that we selected six seed regions for analysis, we also employed a stringent statistical significance in the two-sample *t*-tests. In this case, the height threshold was divided by the number of seed regions (FDR, *p* < 0.05/6).

We also performed regression analyses for rsFCs using the raw scores of PVFT collected from all participants before and after the intervention. In this analysis, regions that showed significant correlations between the rsFCs with seed regions and a difference in the individual PVFT scores between the pre- and post-intervention periods (i.e., post- minus pre-intervention) as an independent variable were explored at the whole-brain level. This analysis enabled us to identify regions that modulated the increase in the score by interacting with the left inferior and middle frontal gyri as seed regions. Participants’ age, sex, and educational level were also included as covariates in the analysis. The threshold at the cluster level was corrected for whole-brain multiple comparisons (FDR, *p* < 0.05).

## Results

**Fig. 1:**
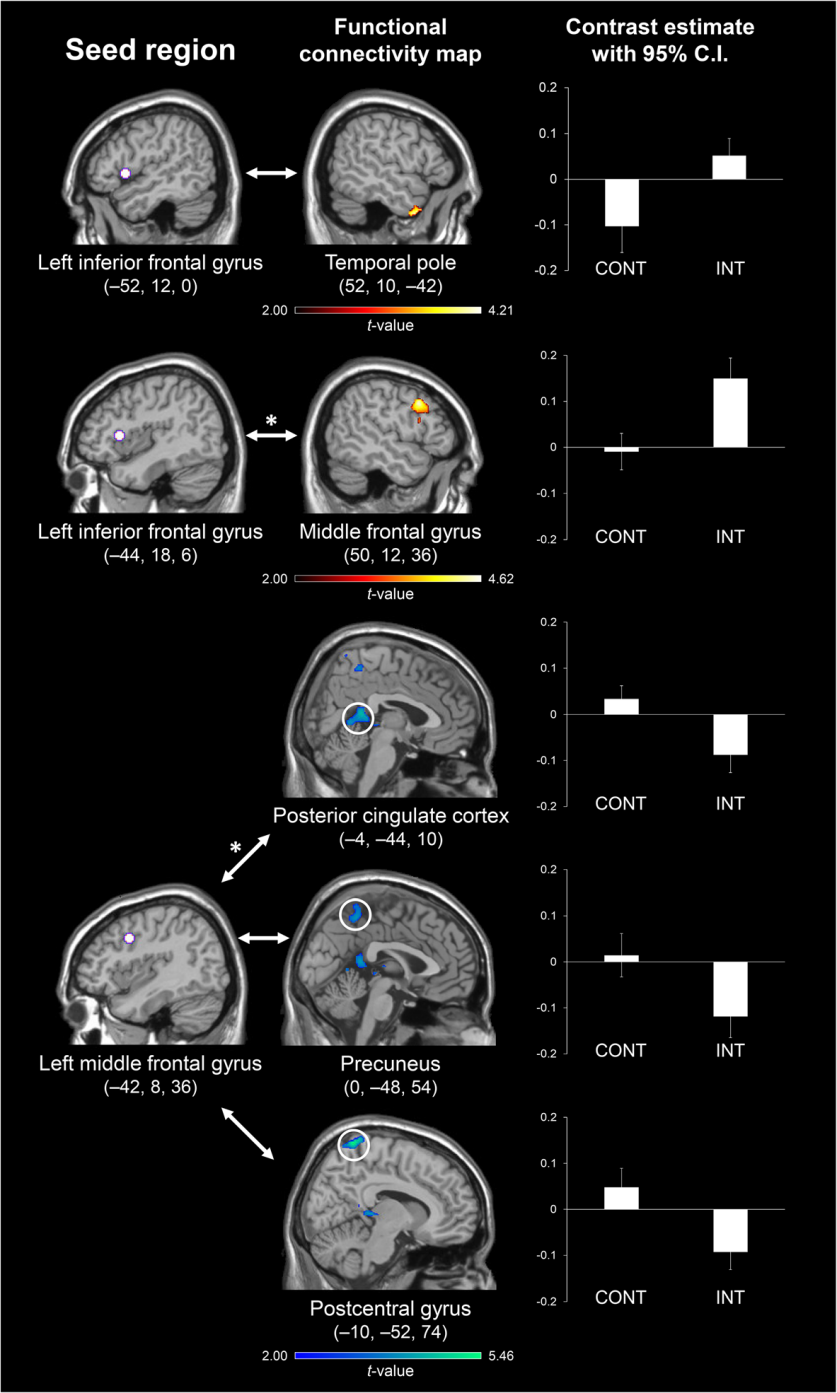
Functional connectivity showing significant group differences. Regions showing significantly greater rsFC with the left inferior frontal gyrus seed centered at (− 52, 12, 0) and (− 44, 18, 6) in INT than in CONT were identified in the temporal pole and middle frontal gyrus, respectively (red). In contrast, regions showing significantly lower rsFC with the left middle frontal gyrus seed centered at (− 42, 8, 36) in INT than in CONT were identified in the posterior cingulate cortex, precuneus, and postcentral gyrus (blue). The bar graphs represent mean contrast estimates within the clusters and error bars represent 95% C.I. Asterisks indicate the survived functional connectivity values even when a stricter height threshold was adopted to correct for whole-brain multiple comparisons (FDR, *p* < 0.05/6). rsFC, resting-state functional connectivity; INT, the intervention group; CONT, the control group; C.I., confidence interval; FDR, false discovery rate

In the present study, the strength of rsFC with the left inferior and middle frontal gyri as seed regions was compared between INT and CONT using two-sample *t*-tests. As illustrated in Fig. 1, significantly higher rsFC was identified in INT than in CONT between the left inferior frontal gyrus seed centered at (− 52, 12, 0) and the temporal pole, and between the left inferior frontal gyrus seed centered at (− 44, 18, 6) and the middle frontal gyrus (FDR, *p* < 0.05). In contrast, two-sample *t*-tests demonstrated that there was significantly lower rsFC in INT than in CONT between the left middle frontal gyrus seed centered at (− 42, 8, 36) and the posterior cingulate cortex, precuneus, and postcentral gyrus (FDR, *p* < 0.05). We did not identify any regions where rsFC was significantly different between INT and CONT with other seeds (FDR, *p* < 0.05). Even when a stringent height threshold was used for whole-brain multiple comparisons, there were rsFC values that remained significant, including the rsFCs between the left inferior frontal gyrus seed centered at (− 44, 18, 6) and the middle frontal gyrus, and between the left middle frontal gyrus seed centered at (− 42, 8, 36) and the posterior cingulate cortex (FDR, *p* < 0.05/6). The findings from the two-sample *t*-tests are summarized in Table 2.

**Table 2:** Regions showing significant group differences in functional connectivity with seed regions

Seed region	Direction	Region	MNI coordinates			Voxel	Z value
			x	y	z		
Left inferior frontal gyrus (−52, 12, 0)	INT > CONT	Temporal pole	52	10	−42	284	3.91
			44	6	−40		3.78
			46	14	−38		3.71
Left inferior frontal gyrus (−44, 18, 6)	INT > CONT	Middle frontal gyrus*	50	12	36	404	4.23
			46	24	30		3.90
			54	12	18		3.81
Left middle frontal gyrus (−42, 8, 36)	INT < CONT	Posterior cingulate cortex*	−4	−44	10	545	4.23
			14	−28	4		3.89
			−4	−54	2		3.85
		Precuneus	0	−48	54	162	3.77
			4	−50	66		3.57
			6	−60	54		3.19
		Postcentral gyrus	−10	−52	74	179	4.87
			−6	−60	70		3.92

Asterisks represent the survived functional connectivity values even when a stricter height threshold was employed to correct for whole-brain multiple comparisons (FDR, *p* < 0.05/6)

*Abbreviations*: *INT* the intervention group, *CONT* the control group, *MNI* Montreal Neurological Institute, *FDR* false discovery rate

For further analysis, we conducted regression analyses, using the difference in the PVFT scores between the pre- and post-intervention periods for all participants. The mean of the difference in the PVFT scores between the pre- and post-intervention was 0.82 (SD 3.51). We confirmed that there was a significant difference in the PVFT scores between the groups after the intervention (*t* [*df* = 59] = 3.16, *p* < 0.01, *Cohen’s d* = 0.81) but not before the intervention (*t* [*df* = 59] = 0.91, *p* = 0.36, *Cohen’s d* = 0.23; see Table 1). The regression analyses showed that the increased PVFT score was significantly correlated with increased rsFC between the left inferior frontal gyrus seed centered at (− 52, 12, 0) and the temporal pole. The rsFC with the other two seeds did not positively correlate with increased PVFT score in any region. No regions showed negative correlations between the rsFC with any seeds and the increased PVFT score. The regression analysis findings are summarized in Table 3.

**Table 3:** Regions showing positive correlation between functional connectivity with seed regions and increased PVFT score

Seed region	Region	MNI coordinates			Voxel	Z value
		x	y	z		
Left inferior frontal gyrus (−52, 12, 0)	Temporal pole	50	6	−22	398	4.26
		48	14	−22		4.12
		46	−6	−12		4.01

*Abbreviations*: *PVFT* phonemic verbal fluency task, *MNI* Montreal Neurological Institute

## Discussion

The present study provides two novel findings. First, significantly higher rsFCs were observed in INT than in CONT between the left inferior frontal gyrus seed and the temporal pole and middle frontal gyrus. Furthermore, the rsFC strength between the left inferior frontal gyrus seed and temporal pole was positively correlated with the increased PVFT score between the pre- and post-intervention periods. Second, significantly lower rsFCs were identified in INT compared to CONT between the left middle frontal gyrus seed and the posterior cingulate cortex, precuneus, and postcentral gyrus. Our findings suggest that the beneficial intervention effect of PICMOR on verbal fluency is related to improved rsFC between the left inferior frontal gyrus and temporal pole. Enhanced rsFC between the left inferior frontal gyrus and middle frontal gyrus and suppressed rsFC between the left middle frontal gyrus and posterior cortical midline structures could also contribute to the enhanced verbal fluency. These findings are discussed further below.

### Regions showing higher rsFC with seed regions in INT than in CONT

Our first major finding was that the rsFCs between the left inferior frontal gyrus as a seed region and the temporal pole and middle frontal gyrus were higher in INT than in CONT (Fig. 1). Significant activation in the temporal pole has been reported in a task-related fMRI study of phonemic verbal fluency [21]. Previous neuroimaging studies have interpreted task-related activation in the anterior temporal lobe, including the temporal pole, in terms of semantic processing [22–24]. This interpretation is supported by evidence from neuropsychological studies of patients with semantic dementia and neurostimulation studies of healthy adults [25–31]. For example, neurostimulation studies using repetitive transcranial magnetic stimulation (rTMS) have consistently shown that the application of rTMS to the left or right temporal pole disrupted semantic processing [32–34]. According to the hub-and-spoke hypothesis, the anterior temporal lobe acts as a hub in semantic processing, which interacts with modality-specific spokes of sensory, motor, and linguistic regions, including the inferior frontal gyrus [26–29]. Supporting this hypothesis, the intrinsic and anatomical connectivity between the anterior temporal lobe and inferior frontal gyrus have been identified in several neuroimaging studies [35–38]. Taken together with previous findings that intrinsic connectivity patterns can predict an individual’s ability to perform cognitive tasks [6], the significantly greater rsFC between the left inferior frontal gyrus and temporal pole in INT compared to CONT suggests that enhanced interactive mechanisms between the two regions at rest contribute to the enhanced ability of INT to access their mental lexicon and retrieve words from their semantic memory. Consistent with this, the results of the regression analyses demonstrated that the rsFC strength between the left inferior frontal gyrus and temporal pole positively correlated with the increased PVFT score between the pre- and post-intervention periods.

Significant activation in the middle frontal gyrus has been identified in several task-related fMRI studies using PVFT [39–45]. Activation in the dorsolateral prefrontal cortex has also been observed in several neuroimaging meta-analyses of the N-back task [46], subsequent memory task [47], Go-NoGo task [48, 49], and Stroop task [50]. All these tasks require executive control processes for successful performance, including retaining various items and task rules in the working memory and/or suppressing inappropriate responses. Thus, the significantly higher rsFC between the left inferior frontal gyrus and middle frontal gyrus in INT compared to CONT suggests that enhanced interactive mechanisms between these regions during the resting-state contribute to the enhanced executive control ability in word production processes, such as retaining earlier verbal responses in working memory and avoiding repetition.

### Regions showing lower rsFC with seed regions in INT than in CONT

The second major finding was that the rsFCs between the left middle frontal gyrus as a seed region and posterior cortical midline structures were lower in INT than in CONT (Fig. 1). Posterior cortical midline structures, including the posterior cingulate cortex, have been shown to deactivate during PVFT [41, 42, 44, 45, 51]. This area has been proposed as a core component of the default mode network [52, 53] (see Additional file 1), which shows greater activation during the resting-state than during the performance of attention-demanding cognitive tasks [54–57]. Although the precise role of the default mode network is still a matter of debate, one possible explanation for activation in the default mode network is that it could involve spontaneous thoughts, i.e., mind-wandering, daydreaming, or task-irrelevant thoughts [58–60]. Given that successful performance in PVFT may rely on the suppression of task-irrelevant thoughts, it is reasonable to suppose that suppressed interactive mechanisms between the left middle frontal gyrus and the default mode network in INT compared with CONT at resting-state contribute to the enhanced ability of INT to suppress spontaneous thoughts during word production.

Results from the regression analyses showed that there were no significant correlations between the rsFC strength for the left middle frontal gyrus seed with the default mode network and the difference in the PVFT score between the pre- and post-intervention periods. This suggests that the suppressed interactive mechanisms between these regions were not directly associated with the beneficial intervention effect of PICMOR on verbal fluency. This could be interpreted as evidence of increased attentional ability. Taken together with previous findings that anticorrelation of the default mode network with task-positive regions was associated with successful task performance [61, 62], the anticorrelated rsFC between the left middle frontal gyrus and the default mode network might reflect increased attentional ability that could indirectly contribute to the beneficial intervention effect on verbal fluency.

### Limitations and future directions

The present study has several limitations. First, any differences in rsFCs between INT and CONT cannot be entirely attributed to the intervention effects because we lack comparable rsfMRI data from the pre-intervention period. In this pilot study, we cannot make any definitive conclusions because it is possible that the two groups intrinsically differed in the functional networks of the brain regions involved in verbal fluency even before the intervention. However, taken together with the behavioral evidence, which showed there was no significant difference in the PVFT score between INT and CONT before the intervention (see Table 1), it is unlikely that our present rsfMRI findings reflect such an intrinsic difference in the brain network between the groups. To obtain a more definitive conclusion, future research will need to obtain rsfMRI data before and after the intervention.

Second, we cannot accurately identify the components in the PICMOR program that contributed to the beneficial intervention effect on verbal fluency. During group conversations, participants in INT received repeated training to make a speech within a limited time (i.e., 1 min), flexibly ask and answer questions, intentionally store and manipulate information to ask questions, and suppress the interruption of other members. In contrast, those in CONT did not receive such training. Given the difference in training demands to exercise executive functions between the two groups, it is not surprising that a significantly larger enhancement in the PVFT score occurred for INT compared to CONT. However, we cannot identify which components in the training contributed to the beneficial intervention effect of PICMOR on verbal fluency. Further investigation is required to clarify this relationship.

Third, our study may have been limited by selection bias for seed regions. In the present study, we applied a seed-based functional connectivity analysis to rsfMRI data. While this approach enabled us to explore rsFC with seed regions at the whole-brain level, it inevitably resulted in selection bias for the seed regions and, therefore, provides a limited amount of information on rsFC across the whole-brain. However, we had valid reasons for selecting the left inferior and middle frontal gyri as seed regions, i.e., we previously found a significantly larger enhancement in the PVFT score in INT than in CONT [4], and there is strong evidence from neuropsychological and neuroimaging studies that the left inferior and middle frontal gyri play a critical role in verbal fluency [10–15]. Therefore, we believe that the seed-based approach was still an effective way to achieve the aim of the present study. A data-driven approach, including whole-brain multivariate pattern and independent component analyses [63], could be used in future research to overcome the disadvantage of the seed-based approach.

The final limitation is related to the correction for multiple comparisons in the rsfMRI data analysis. Specifically, in the present study, the correction for the whole-brain multiple comparisons was performed using FDR, *p* < 0.05. However, because we selected six seed regions to compare INT and CONT, adopting a stricter statistical significance might be more accurate. When we employed the height threshold divided by the number of seed regions in the two-sample *t*-tests, some rsFC values survived, including the rsFCs between the left inferior frontal gyrus seed centered at (− 44, 18, 6) and the middle frontal gyrus, and between the left middle frontal gyrus seed centered at (− 42, 8, 36) and the posterior cingulate cortex. However, other rsFC values failed to achieve stricter statistical significance. Despite this limitation, this preliminary report successfully achieved its exploratory purpose and provided candidates for rsFCs, reflecting the beneficial effects of PICMOR on the brain network responsible for verbal fluency.

In future research, it is important to elucidate the neural mechanisms by which verbal fluency is enhanced by interventions, given that there is some behavioral evidence that verbal fluency can be trained [64] (see Additional file 1). This pilot study provides a basis for such future research. In our previous RCT, the ability of verbal fluency was assessed by a neuropsychological test in an experimental setting [4], as in other intervention studies [2]. However, little is known about how interventions affect cognitive task performance in real-world situations. Future studies that compare verbal fluency in day-to-day conversations between the pre- and post-intervention periods could identify how the intervention effects identified in experimental settings could be extended to real-world situations.

## Conclusions

In the present study, we investigated the differences in the functional networks of the brain regions involved in verbal fluency at resting-state between INT and CONT after the intervention period. Results showed that the rsFC between the left inferior frontal gyrus and the temporal pole was higher in INT than in CONT, and that the rsFC strength was positively correlated with the increased PVFT score between the pre- and post-intervention periods. The rsFC between the left inferior frontal gyrus and the middle frontal gyrus was also higher in INT than in CONT. In contrast, the rsFCs between the left middle frontal gyrus and the posterior cortical midline structures were lower in INT than in CONT. These findings suggest that the beneficial intervention effect of PICMOR on verbal fluency is related to increased rsFC of the left inferior frontal gyrus with the semantic-related temporal pole. The increased rsFC between the left inferior frontal gyrus and executive control-related middle frontal gyrus, as well as the suppressed rsFC between the left middle frontal gyrus and posterior cortical midline structures, could also contribute to the enhancement of verbal fluency. No definitive conclusions can be drawn because our study lacked comparable rsfMRI data from the pre-intervention period. However, this pilot study achieved its exploratory purpose and provided candidates for rsFCs, reflecting the neural mechanisms that underlie the beneficial intervention effect of PICMOR on verbal fluency.

## Supplementary Information

**Additional file 1.** A supplementary analysis for rsfMRI data and a follow-up study.

## Data Availability

The datasets generated and/or analyzed during the current study are not publicly available because a joint research agreement is required for data sharing, but are available from the corresponding author on reasonable request.

## Abbreviations

PICMOR: Photo-Integrated Conversation Moderated by Robots
RCT: Randomized controlled trial
PVFT: Phonemic verbal fluency task
INT: The intervention group
CONT: The control group
rsfMRI: resting-state functional magnetic resonance imaging
rsFC: resting-state functional connectivity
TR: Repetition time
TE: Echo time
FOV: Field of view
BOLD: Blood oxygenation level-dependent
SPM: Statistical Parametric Mapping
MNI: Montreal Neurological Institute
FDR: False discovery rate
SD: Standard deviation
C.I.: Confidence interval
rTMS: repetitive transcranial magnetic stimulation
