# Supplementary material for: Characteristics of resting-state functional connectivity in older adults after the PICMOR intervention program: a preliminary report
Source: BMC Geriatr. 2020 Nov 20;20:486. doi: 10.1186/s12877-020-01892-2 (PMC7678164; doi:10.1186/s12877-020-01892-2)
Supplement: Supplementary file 1 — Additional file 1. A supplementary analysis for rsfMRI data and a follow-up study. [file 12877_2020_1892_MOESM1_ESM.docx]

**Supplementary Information**

**Characteristics of Resting-State Functional Connectivity in Older Adults after the PICMOR Intervention Program: A Preliminary Report**

Hikaru Sugimoto, Toshikazu Kawagoe, and Mihoko Otake-Matsuura

# A supplementary analysis for rsfMRI data

We interpreted the results of the posterior cingulate cortex, which showed lower rsFC with the left middle frontal gyrus in INT than in CONT, in terms of the default mode network. To confirm the validity of this, we conducted a seed-based analysis of the rsfMRI data, employing a cluster of the posterior cingulate cortex (x = −4, y = −44, z = 10; k = 545) as a seed region. As shown in Supplementary Fig. 1, the posterior cingulate cortex had significant rsFCs with the medial prefrontal cortex and lateral parietal cortex (FDR, *p* < 0.05), which has been associated with the default mode network [1]. Our interpretation is supported by these supplementary findings.


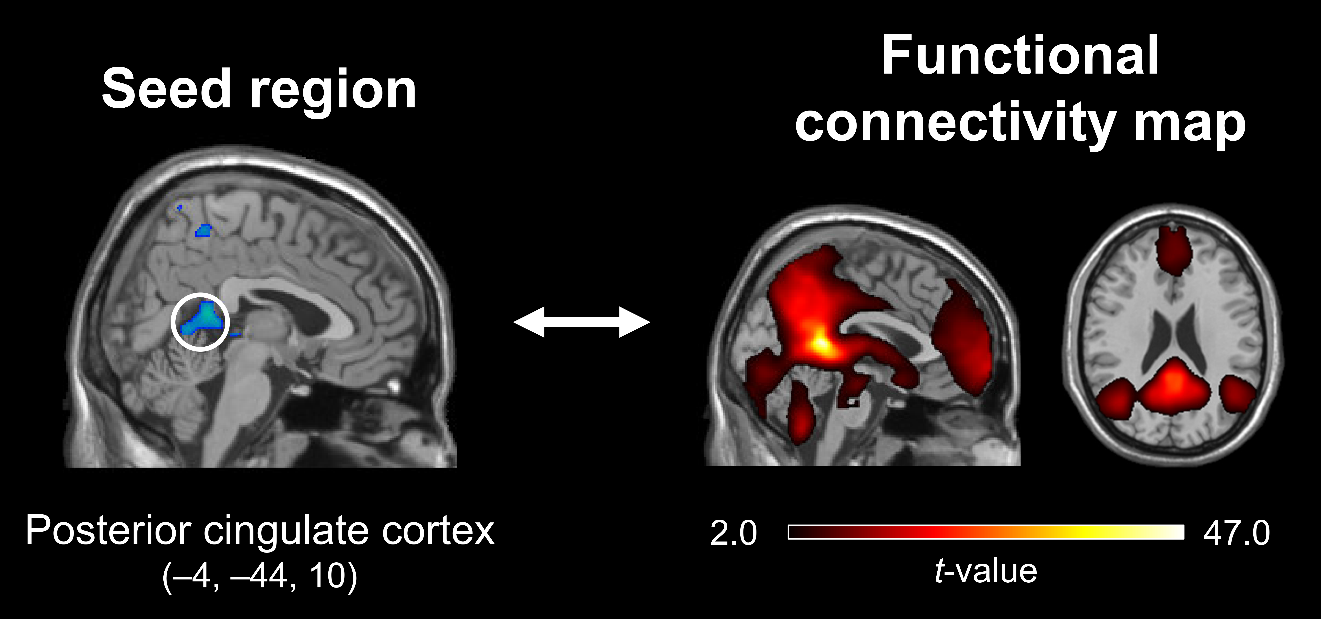


**Supplementary Fig. 1** Functional connectivity with the posterior cingulate cortex

# ****A follow-up study****

To address the sustainability of the beneficial intervention effect on verbal fluency identified in our previous RCT study [2], we conducted a follow-up experiment almost 1 year after the intervention period using PVFT, although it is out of the scope of this pilot rsfMRI study. Among the sixty-one participants in the rsfMRI experiment (31 and 30 participants in INT and CONT, respectively), a total of fifty-six participants, including 28 participants in INT (14 females and 14 males; 17 people with education for 13 years and more; age, mean ± SD = 72.82 ± 3.60 years) and 28 participants in CONT (16 females and 12 males; 15 people with education for 13 years and more; age, mean ± SD = 71.89 ± 2.77 years), participated in this follow-up experiment. There were no significant differences in age (*t* [*df* = 54] = 1.08, *p* = 0.28), sex (*χ^2^* [*df* = 1, n = 56] = 0.07, *p* = 0.79), and educational level (*χ^2^* [*df* = 1, n = 56] = 0.07, *p* = 0.79) between the two groups. Also, there was no significant difference in the time span from the last day of the intervention period to the day of the follow-up experiment between INT (mean ± SD = 54.08 ± 0.55 weeks) and CONT (mean ± SD = 54.12 ± 0.36 weeks) (*t* [*df* = 54] = 0.29, *p* = 0.77). We found a significantly larger PVFT score at the follow-up in INT (mean ± SD = 13.64 ± 4.12) than in CONT (mean ± SD = 11.36 ± 3.86) (*t* [*df* = 54] = 2.14, *p* < 0.05, *Cohen's d* = 0.57). Given the evidence from another intervention study that showed a remarkable improvement in the PVFT score 12 weeks after the intervention period [3], our finding extended the previous findings by demonstrating that the beneficial effect on verbal fluency could last for a longer period.

## Abbreviations

rsfMRI: resting-state functional magnetic resonance imaging; rsFC: resting-state functional connectivity; INT: the intervention group; CONT: the control group; FDR: false discovery rate; RCT: randomized controlled trial; PVFT: phonemic verbal fluency task; SD: standard deviation.

## References

1. Raichle ME. The brain's default mode network. Annu Rev Neurosci. 2015;38:433-47.

2. Otake-Matsuura M, Tokunaga S, Watanabe K, Abe MS, Sekiguchi T, Sugimoto H, et al. Photo-integrated conversation moderated by robots for cognitive health in older adults: a randomized controlled trial. Preprint at https://doi.org/10.1101/19004796 (2019).

3. Dodge HH, Zhud J, Mattek NC, Bowman M, Ybarrae O, Wild KV, et al. Web-enabled conversational interactions as a method to improve cognitive functions: results of a 6-week randomized controlled trial. Alzheimers Dement (N Y). 2015;1(1):1-12.
